# Downregulation of Extracellular Matrix and Cell Adhesion Molecules in Cumulus Cells of Infertile Polycystic Ovary Syndrome Women With and Without Insulin Resistance

**DOI:** 10.22074/cellj.2019.5576

**Published:** 2018-11-18

**Authors:** Fatemeh Hassani, Shahrbanoo Oryan, Poopak Eftekhari-Yazdi, Masood Bazrgar, Ashraf Moini, Nahid Nasiri, Ali Sharifi-Zarchi

**Affiliations:** 1 Department of Animal Biology, Faculty of Biological Sciences, Kharazmi University, Tehran, Iran; 2 Department of Embryology, Reproductive Biomedicine Research Center, Royan Institute for Reproductive Biomedicine, ACECR, Tehran, Iran; 3 Department of Genetics, Reproductive Biomedicine Research Center, Royan Institute for Reproductive Biomedicine, ACECR, Tehran, Iran; 4 Department of Endocrinology and Female Infertility, Reproductive Biomedicine Research Center, Royan Institute for Reproductive Biomedicine, ACECR, Tehran, Iran; 5 Department of Obstetrics and Gynecology, Arash Women’s Hospital, Tehran University of Medical Sciences, Tehran, Iran; 6 Department of Stem Cells and Developmental Biology, Cell Science Research Center, Royan Institute for Stem Cell Biology and Technology, ACECR, Tehran, Iran

**Keywords:** Cumulus Cells, Extracellular Matrix, Gene Expression, Insulin Resistance, Polycystic Ovary Syndrome

## Abstract

**Objective:**

The extracellular matrix (ECM) of the cumulus oocyte complex (COC) is composed of several molecules that have different roles during follicle development. This study aims to explore gene expression profiles for ECM and cell adhesion molecules in the cumulus cells of polycystic ovary syndrome (PCOS) patients based on their insulin sensitivity following controlled ovarian stimulation (COS).

**Materials and Methods:**

In this prospective case-control study enrolled 23 women less than 36 years of age who participated in an intracytoplasmic sperm injection (ICSI) program. Patients were subdivided into 3 groups: control (n=8, fertile women with male infertility history), insulin resistant (IR) PCOS (n=7), and insulin sensitive (IS) PCOS (n=8). We compared 84 ECM component and adhesion molecule gene expressions by quantitative real-time polymerase chain reaction array (qPCR-array) among the groups.

**Results:**

We noted that 21 of the 84 studied genes differentially expressed among the groups, from which 18 of these genes downregulated. Overall, comparison of PCOS cases with controls showed downregulation of extracellular matrix protein 1 (*ECM1*); catenin (cadherin-associated protein), alpha 1 (*CTNNA1*); integrin, alpha 5 (*ITGA5*); laminin, alpha 3 (LAMA3); laminin, beta 1 (*LAMB1*); fibronectin 1 (*FN1*); and integrin, alpha 7 (*ITGA7*). In the IS group, there was upregulation of ADAM metallopeptidase with thrombospondin type 1 motif, 8 (*ADAMTS8*) and neural cell adhesion molecule 1 (*NCAM1*) compared with the controls (P<0.05).

**Conclusion:**

Downregulation of ECM and cell adhesion molecules seem to be related to PCOS. Gene expression profile alterations in cumulus cells from both the IS and IR groups of PCOS patients seems to be involved in the composition and regulation of ECM during the ovulation process. This study highlights the association of ECM gene alteration as a viewpoint for additional understanding of the etiology of PCOS.

## Introduction

Polycystic ovary syndrome (PCOS) is a frequent endocrinopathic condition among reproductive aged women with a prevalence of 8-12% (1). According to the Rotterdam ESHRE/ASRM Consensus, diagnostic criteria for PCOS include oligo/anovulation, hyperandrogenism, and polycystic ovaries (detected by sonography) (2). Although the etiology of PCOS is uncertain, there is a confirmed familial and genetic basis for PCOS (3). The consequential complications of PCOS are follicular maturation arrest and insulin resistance (4, 5). Insulin resistance is defined as the impaired insulin ability to maintain glucose homeostasis, which leads to an increase in insulin levels in the bloodstream (6). The role of insulin resistance in the pathogenesis of PCOS is uncertain, but studies lend support to the hypothesis that insulin plays an important role in regulating the response of human granulosa cells to gonadotropins (7). Hyperinsulinemia is a condition that damages oocyte developmental competence, resulting in reduced rates of fertilization, embryonic development, and implantation in obese PCOS patients (8).

Folliculogenesis needs communication between the oocyte and surrounding somatic cells (9, 10). These somatic cells comprise two populations, specialized layers of flattened granulosa cells which line the antrum of follicles and a specified type of granulosa cells called cumulus cells which surround the oocyte in the preovulatory follicle. Cumulus cells undergo “cumulus expansion”, a process that requires these cells to form new ECM that binds the oocyte and cumulus cells together (5, 11). This process enables the oocyte to resume maturation. A surge of luteinizing hormone (LH) is necessary to initiate ovulation (5). 

The extracellular matrix (ECM) of the cumulus oocyte complex (COC) is composed of several molecules with varying roles such as differentiation, division, cell death, and migration. Interestingly, all of these roles are associated with follicle development. Appropriate formation of the expanded cumulus matrix is critical for ovulation. Successful follicular rupture and fertilization is sensitive to perturbations in the composition and functional capacity of the cumulus matrix (11). The backbone of the expanded cumulus matrix is hyaluronic acid (HA), a large disaccharide chain common to numerous ECM. Synthesis of HA requires glucose. Glucose uptake and glycolytic activity in cumulus cells are markedly stimulated by the LH surge in rodents, cows, and humans. During oocyte maturation, there is an increase in glucose flux in the COC. The basement membrane that surrounds the granulosa layers of all follicles is composed of type I collagen, fibronectin, and laminin (12).

Proteoglycans such as versican (VCAN) are produced primarily by mural granulosa cells and rapidly incorporate into developing cumulus matrix. This suggests that VCAN binds to HA through its link module and is another organizer of the COC matrix structure. Deregulation of ECM matrix compartment genes during follicular development is important in the pathogenesis of PCOS (13).

Follicular growth and rupture, as well as early luteal formation, partially occur through the action of matrix metalloproteinase (MMPs) and their inhibitors. The MMP system is involved in connective tissue remodeling processes throughout the body. This system comprises both proteolytic enzymes and their associated inhibitors. MMPs have a potent ability to bind and cleave gelatin and act to degrade major constituents of basement membranes that include type IV collagen, laminin, and fibronectin. In the ovary, MMPs and their inhibitors are hypothesized to play a critical role in ECM remodeling associated with ovulation, luteal formation, and regression (14).

Follicular development and ovulation are dynamic processes that need broad tissue remodeling. Previous studies have reported the abnormal turnover of ovarian ECM components that lead to development of PCOS (15). 

In the present study, we assessed the gene expression profiles for ECM and adhesion molecules in the cumulus cells of infertile PCOS patients based on their insulin sensitivity following ovarian stimulation with a gonadotropin-releasing hormone (GnRH) antagonist protocol. We reported downregulation of ECM and cell adhesion molecules as a probable etiology of PCOS infertility.

## Materials and Methods

### Patient selection

The Ethics Committee at Royan Institute approved this prospective case-control study (No. EC/93/1078). All participants gave informed consent prior to inclusion in the study. We ensured the confidentiality of patients’ identities this research by data anonymization during analysis. This research did not incur any additional costs to the patients, nor did it affect their treatment in any way. Study participants comprised 23 women, less than 36 years of age, who underwent intracytoplasmic sperm injection (ICSI) and were not affected by thyroid disorders, diabetes, or ovarian hyperstimulation syndrome (OHSS). We allocated 15 PCOS patients previously diagnosed by the Rotterdam 2004 criteria whose partners had normal spermogram results (2) to one of two groups, insulin resistant (IR) or insulin sensitive (IS), based on fasting insulin (FI, cutoff: 12 mU/L) levels and the homeostasis model assessment of insulin resistance (HOMA-IR, cutoff: 2.57). We calculated HOMA-IR as follows: [(fasting serum insulin [mU/L]×fasting serum glucose [mmol/L])/22.5] (16).

The IR group consisted of 7 PCOS patients (FI=12 mU/L, HOMA-IR:=2.57). The IS group consisted of 8 patients (FI:<12 mU/L; HOMA-IR<2.57). The control group consisted of 8 healthy, normal ovulatory fertile women with male infertility history.

### Stimulation protocol

Controlled ovarian stimulation (COS) was initiated from the third day of the cycle. Patients received regular, daily subcutaneous (SC) injections of recombinant follicle-stimulating hormone (rFSH, Gonal-F, Serono, Switzerland). We adjusted the starting dose of rFSH according to each patient’s response as measured by transvaginal ultrasonography, antral follicle count (AFC), estradiol (E2) level, and anti-Müllerian hormone (AMH). Once the ovarian follicles reached 12 mm in diameter, patients received SC injections of a GnRH antagonist, cetrorelix (Cetrotide®, Merck Serono, Germany). The protocol consisted of daily Cetrotide® SC injections until the criteria for human chorionic gonadotropin (hCG) administration was met. When more than 3 follicles reached diameters of at least 18 mm and E2 levels of 1000-4000 pg/mL, each patient received an intramuscular (IM) injection of 10000 IU of hCG (Pregnyl®, Organon, Netherlands) or SC injection of 250 µg Ovidrel (Merck Serono, Germany).

### Isolation of cumulus cells 

Following oocyte pick-up, the COCs were washed 3-5 times in G-IVFTM medium (Vitrolife, Sweden) to remove blood and excess cells. After washing, the COCs were placed in a CO_2_ incubator at 37°C for 2 hours in G-IVFTM (Vitrolife, Sweden). Oocyte denudation was performed with 80 IU of hyaluronidase, (Sigma, USA) (17). Immediately after oocyte denudation, cumulus cells were washed with phosphate-buffered saline (PBS) and we added RNA protect, after which the cells were snap frozen in liquid nitrogen and stored at -80°C until RNA extraction. Cumulus cells were collected from metaphase II oocytes (MII). M.. oocytes were fertilized by ICSI within 10 minutes after denudation, and then incubated until transfer. The regular fertilization rate was controlled (16-20 hours after ICSI). Based on our laboratory standards, embryos were graded at the pronuclear (16-20 hours) and cleavage (48-72 hours) stages (18, 19). We selected 1-2 embryos for transfer based on the embryos’ grades, patient age, and previous assisted reproductive technology (ART) cycles.

### Purification and preparation of RNA

Total RNA was extracted by a Pico Pure RNA Isolation Kit (Arcturus, USA) and treated with RNase-free DNase I according to the manufacturer’s instructions. RNA concentration and purity were quantified using a Nanodrop 2000 Spectrophotometer (Thermo, USA).

### Quantitative real-time PCR array

We preamplified 50 ng of total RNA using the RT2 PreAMP cDNA Synthesis Kit (Qiagene, USA) in a 12-cycle multiplex PCR for all genes of interest. We examined the same set of genes in the 3 study groups. Quantitative real-time PCR array (qPCR-array) was performed using the Human Extracellular Matrix & Adhesion Molecules RT2 Profiler PCR Array (Qiagene, USA). These SYBR Green-based arrays were designed as one sample/one 96-well plate using primers for a preset list of genes that included 84 ECM and adhesion molecule genes in addition to 12 control wells. Only experiments that passed the PCR array run quality control were included in the data analyses. Briefly, cDNA volumes were adjusted to 2.5 ml with RT2 Real-Time SYBR Green/ROX PCR Master Mix (Qiagene, USA). A total of 25 µL cDNA mix was added to all wells. Real-time PCR was performed in a StepOnePlus™ instrument (Applied Biosystems, USA). 

### Bioinformatics and statistical analysis 

Relative gene expressions were calculated by the 2^-ΔΔCt^ method. Ct indicated the cycle threshold, the fractional cycle number where the fluorescent signal reached the detection threshold. The normalized ΔCt value of each sample was calculated using reference genes with a Ct variation less than one among all experiments. Reference genes included beta-2 microglobulin (B2M); ribosomal protein, large, P0 (*RPLP0*); hypoxanthine phosphoribosyltransferase 1 (*HPRT1*); actin, beta (*ACTB*); and glyceraldehyde 3-phosphate dehydrogenase (*GAPDH*). The statistical significance of differentiallyexpressed genes (DEG) was measured by the two-tailed t test. Two-sided P<0.05 were considered significant.

Visualization of the biological function network of DEG was performed using a search tool for the retrieval of interacting genes/proteins (STRING; http://string-db.org/), an online functional protein interaction network. 

## Results

Clinical parameters including age, LH/FSH ratio, body mass index (BMI), and concentration of fasting blood glucose did not significantly differ among the three groups (P>0.05). Duration of infertility tended to be longer in IR patients as compared with the control group (P=0.097); however, it was not different considering other comparisons (P>0.05). LH concentration tended to be higher in IS patients than the control group (P=0.079), but it did not differ between other groups (P>0.05).

The number of follicles and oocytes collected per patient did not significantly differ between groups (P>0.05). Number of MII oocytes was greater in the control group compared with IS (P=0.015) and IR (P=0.048) groups. However, number of MII oocytes was not different between IS and IR groups (P>0.05). There was no difference in the fertilization rate of oocytes among the three groups. Both the IR and IS groups had significantly lower numbers of good quality cleavage stage embryos compared to the control group (P<0.01). The number of transferred embryos did not differ between groups (P>0.05, Tables1, 2).

**Fig.1:**
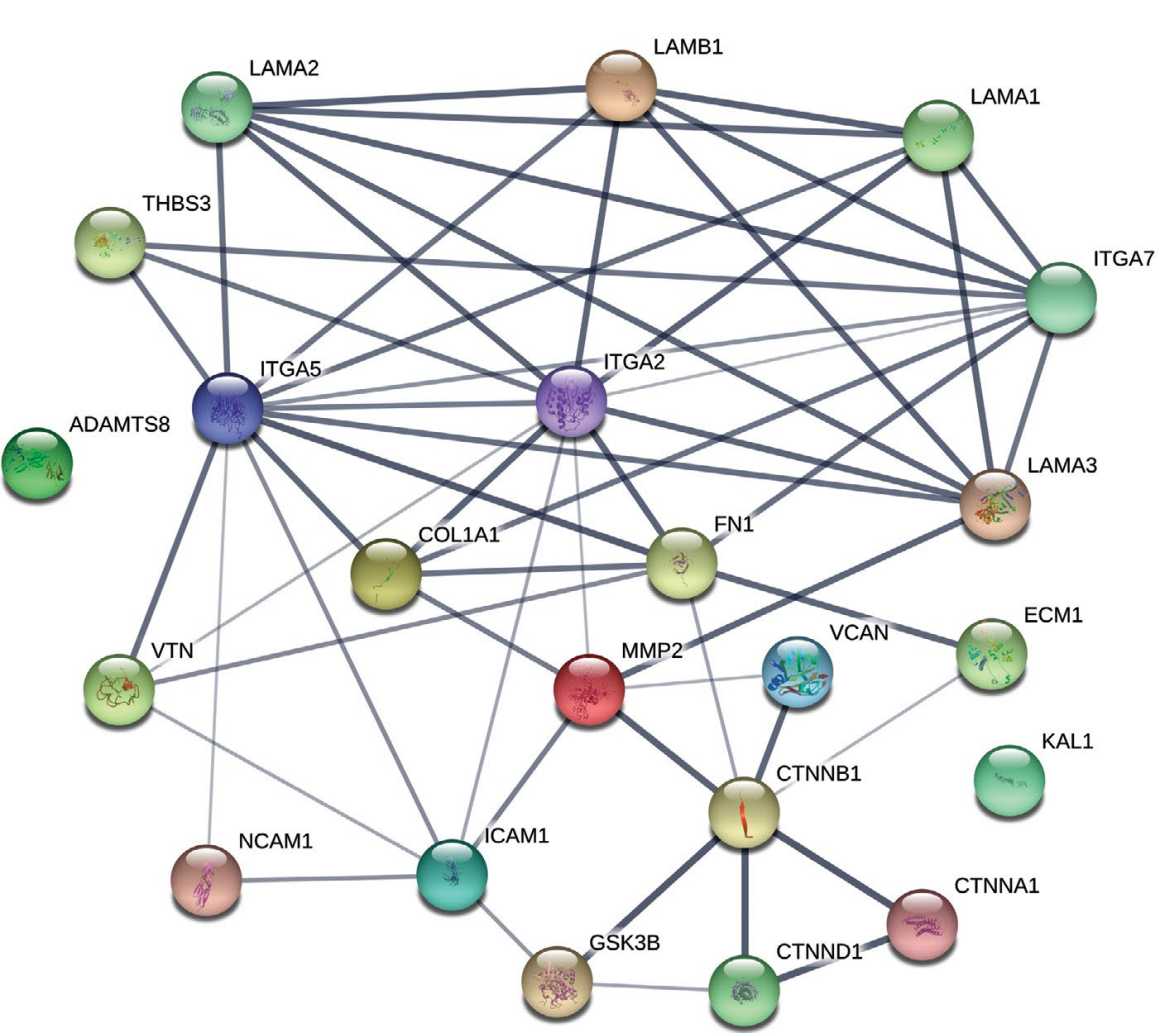
Protein-protein interaction network of respective proteins to differentially expressed genes (DEG) in cumulus cells from among the groups. Thicknesses of interactions show confidence levels according to the STRING database.

We analyzed the expression profiles of 84 genes relatedto the ECM protein and adhesion molecule pathway. Of thefive reference genes, *B2M, RPLP0* and *HPRT1*, with a Ct variation less than one among all experiments, were chosenfor normalization. Table 3 shows the fold differences for DEG among the groups (P<0.05). In the IS group, ADAMmetallopeptidase with thrombospondin type 1 motif, 8(*ADAMTS8*) and neural cell adhesion molecule 1 (*NCAM1*) upregulated whereas integrin, alpha 2 (*ITGA2*); collagen, type I, alpha 1 (*COL1A1*); fibronectin 1 (*FN1*); integrin, alpha 7 (*ITGA7*); and matrix metallopeptidase 2 (*MMP2*) downregulated compared to the control group. Extracellularmatrix protein 1 (*ECM1*) and integrin, alpha 5 (*ITGA5*) downregulated in the IR group compared to the controlgroup (P=0.030 and P=0.052, respectively). A comparisonof the IR group with the IS group showed downregulationof catenin (cadherin-associated protein), beta 1(*CTNNB1*); catenin (cadherin-associated protein), delta 1 (*CTNND1*); intercellular adhesion molecule 1 (*ICAM1*); Kallmann syndrome 1 sequence (*KAL1*); laminin, alpha 1 (*LAMA1*); laminin, alpha 2 (*LAMA2*); VCAN, and vitronectin (*VTN*) along with upregulation of *ITGA2*. Comparison between allPCOS patients to controls showed downregulation of *ECM1*; catenin (cadherin-associated protein), alpha 1 (*CTNNA1*); *ITGA5*; laminin, alpha 3 (*LAMA3*); laminin, beta 1 *(LAMB1); FN1;* and *ITGA7*. Figure 1 shows the network of the respective proteins of DEG. Although this figure does not show a mechanism behind our observation, it shows interactions among these genes. Hence, they are not isolated, independent genes; rather, their produced proteins might cooperate as a cluster. 

**Table 1:** Clinical parameters for control and PCOS patients


Patients (n)	Control group	IR group	IS group	P value
	n=8	n=7	n=8	

Age (Y)	30.29 ± 2.15	27.75 ± 1.37	26.25 ± 1.13	NS
Duration of infertility (Y)	3.14 ± 1.23	6.75 ± 1.25	5.31 ± 0.96	NS
BMI (kg/m^2^)	23.60 ± 1.47	27.61	27.18 ± 1.98	NS
FSH (U/L)	5.37 ± 0.59	7.55 ± 1.56	6.51 ± 0.75	NS
LH (U/L)	5.10 ± 0.59	6.19 ± 0.7	8.12 ± 1.24	NS
LH/FSH	1.14 ± 0.32	1.07 ± 0.26	1.27 ± 0.17	NS
Fasting glucose (mg/dl)	93.14 ± 2.87	91.75 ± 1.42	93.38 ± 3.56	NS


Variables are presented as Mean ± SE. P values determined by analyzed generalized linear model(GLM) procedure with significance level of P<0.05. PCOS; Polycystic ovary syndrome, BMI; Body mass index, FSH; Follicle-stimulating hormone, LH; Luteinizing hormone, IS; Insulin sensitive, IR; Insulin resistant, NS; Not significant.

**Table 2:** Cycle characteristics and IVF/ICSI outcomes in controls compared to PCOS patients


Variables	Control group	IR group	IS group	P value
	n=8	n=7	n=8	

Number of follicles	14.14 ± 1.55	18.25 ± 1.67	14.75 ± 2.30	NS
Number of oocytes retrieved	16.43 ± 1.45	13.00 ± 1.68	12.38 ± 1.92	NS
Number of MII oocytes	15.43 ± 1.23^a^	10.88 ± 1.27^b^	9.88 ± 1.22^c^	a, b=0.048
				a, c=0.015
Regular fertilization rate, % (# of 2PN/# MII oocytes)	72 (78/108)	69 (54/78)	68 (54/79)	NS
Total embryos	10.14 ± 0.86	7.25 ± 1.25	8.00 ± 1.18	NS
Number of good quality embryos	5.14 ± 0.83^a^	200. ± 0.78^b^	1.62 ± 0.60^c^	a, b=0.016
				a, c=0.005
Number of ET	1.00 ± 0.49	1.75 ± 0.53	1.13 ± 0.44	NS


Variables are presented as Mean ± SE. P values determined with significance level of P<0.05. PCOS; Polycystic ovary syndrome, IVF; In vitro fertilization, ICSI; Intracytoplasmic sperm injection, IS; Insulin sensitive, IR; Insulin resistant, MII; Metaphase II oocytes, NS; Not significant, 2PN; Two pronuclei, ET; Embryos transferred, ^a, b^ ; Statistically significant differences between IR patients vs. controls, and ^a,c^ ; Statistically significant differences between IS patients vs. controls.

**Table 3:** Differentially expressed gene fold differences among groups


Gene symbols	IS vs. control	IR vs. control	IR vs. IS	PCOS vs. control	P value

*ADAMTS8*	3.32^a^	0.63	2.26	2.77	a=0.031^∗^
*COL1A1*	0.43^a^	2.21	0.94	0.62	a=0.020^∗^
*CTNNA1*	0.60	0.98	0.58	0.59^d^	d=0.022^∗^
*CTNNB1*	3.78	1.18	0.31^c^	2.19	c=0.013^∗^
*CTNND1*	1.78	0.73	0.41^c^	1.18	c=0.028^∗^
*ECM1*	0.66^a^	0.49^b^	0.73	0.57^d^	a=0.052^†^
					b=0.030^∗^
					d=0.011^∗^
*FN1*	0.51^a^	0.65	1.27	0.57^d^	a=0.004^∗∗^
					d=0.012^*^
*ICAM1*	1.94	0.78	0.4^c^	1.27	c=0.011^∗^
*ITGA2*	0.54^a^	0.88	1.64^c^	0.68	a=0.020^∗^
					c=0.042^∗^
*ITGA5*	0.63	0.58^b^	0.92	0.61^d^	b=0.052^†^
					d=0.033^∗^
*ITGA7*	0.45^a^	0.51	1.14	0.47^d^	a=0.046^∗^
					d=0.017^∗^
*KAL1*	2.09	1.21	0.58^c^	1.62	c=0.002^∗∗^
*LAMA1*	3.96	1.13	0.28^c^	2.20	c=0.028^∗^
*LAMA2*	2.19	0.86	0.39^c^	1.42	c=0.018^∗^
*LAMA3*	0.68	0.52^b^	0.76	0.6^d^	b=0.059^†^
					d=0.022^∗^
*LAMB1*	0.67	0.60	0.90	0.64^d^	d=0.020^∗^
*MMP2*	0.40^a^	1.69	4.21	0.79	a=0.037^∗^
*NCAM1*	2.72^a^	1.64	0.60	2.15	a=0.049^∗^
*THBS3*	0.48^a^	0.77	1.60	0.60	a=0.039^∗^
*VCAN*	3.50	1.01	0.29^c^	1.96	c=0.030^∗^
*VTN*	2.29	1.26	0.55^c^	1.73	c=0.037^∗^


The statistical significance of differentially expressed genes (DEG) was measured by the two-tailed t test. IS; Insulin sensitive, IR; Insulin resistant, PCOS; Polycystic ovary syndrome, *; P<0.05, **; P<0.01, †; statistically marginal difference 0.05<P<0.06. Last column represents P value of comparison between groups, a; IS vs. control, b; IR vs. control, c; IR vs. IS, and d; PCOS vs. control.

## Discussion

In terms of* in vitro* fertilization (IVF)/ICSI outcome, the present study showed that IR might be associated with low oocyte maturity in infertile PCOS women, but this did not affect the regular fertilization rate of oocytes between the 3 groups. According to our data, both the IR and IS groups had significantly lower numbers of good quality embryos compared to the control group. 

The expression pattern of cumulus cells of infertile PCOS patients in an IVF program was studied and compared based on their insulin sensitivity. Differences arise in the expression of genes involved in the composition and regulation of COC ECM. We highlighted the association of ECM and cell adhesion molecule gene alterations in order to understand the etiology of PCOS as a genetically complex disorder. The importance of cumulus cells in the control of oocyte metabolism has been reported (20). Malfunction of these cells might have a role in PCOS pathogenesis (21).

Since the report on insulin hypersecretion by Burghen et al. (22), this disorder has been reported consistently in women with PCOS. There are molecular mechanisms that can elucidate insulin resistance in PCOS patients. It seems that a major contributor to insulin resistance in PCOS patients is a reduction in insulin sensitivity secondary to a defect in insulin signaling (23). Recent *in vitro* studies have revealed differential insulin signaling in human luteinized granulosa cells of PCOS patients with and without insulin resistance (24). According to recent studies, comparison of PCOS patients with controls has shown differential expression of ECM related genes. The studied DEGs associated with O- and N-glycosylation, which is important in ECM components gathering; these mechanisms highlight the key role of ECM components during folliculogenesis (25). Differential expression of ECM and cell adhesion molecules genes were identified in IR versus IS PCOS patients. It seemed that dysregulation of ECM components could associate with defective oocyte maturation, as well as a decrease in embryo quality, even after IVF treatment. 

Among DEG detected in this study, an association with some genes had previously been reported with PCOS, such as *ADAMTS8*; integrin, beta 2 *(ITGB2); CTNNB1*; and cadherin 1 (*CDH1*) (26, 27). 

In the present study, we have observed downregulation of *CTNNB1* and *CTNND1* in IR PCOS patients compared to IS PCOS patients. *CTNNB1*, is a key effector of the canonical Wnt/frizzled (FZD) pathway. *CTNNB1* not only mediates cell-cell adhesion, but also acts as a transcription factor. In the latter context, *CTNNB1* protein is phosphorylated and subsequently degraded by a large multi-protein complex that includes glycogen synthase kinase 3 beta (GSK3ß) (28). Microarray analysis of PCOS ovaries compared to normal ovaries have shown downregulation of genes that encode for components of Wnt signaling (27). In animal studies, disruption of *CTNNB1* expression in granulosa cells is predictive of major changes in granulosa cell performance (29).

We observed downregulation of *VCAN* in IR versus IS patients, which agreed with a recent study that has highlighted a possible role for *VCAN* in ovulatory dysfunction of PCOS patients (30). VCAN is one of the markers of oocyte developmental competence. According to Gebhardt et al. (31), cumulus cells separated from oocytes that led to live birth had significantly elevated *VCAN* expression.

Expression of the *KAL1* gene decreased significantly in IR versus IS patients. A recent study highlighted the role of *KAL1* as one of the ECM components in oocyte maturation (32). In our study, downregulation of *KAL1* in IR versus IS patients interfered with normal oocyte maturation.

We observed downregulation of *MMP2* in the IS group compared to the control group. Curry and Osteen (33) proposed that the MMP system might regulate normal follicular maturation and atresia in order to attain the appropriate number of ovulatory follicles. Recent studies showed that *MMP2* highly expressed during ovulation (34); therefore, downregulation of this gene in PCOS patients could affect normal ovulation.

Insulin resistance can lead to structural alterations in the basal lamina of the insulin-responsive organs. Under the influence of insulin resistance, ovulation mechanisms in the ovaries are impaired and hyperinsulinemia is present prior to anovulation (6, 24). Cumulus cells organize the ECM structure prior to ovulation and provide a microenvironment essential for normal fertilization. In this regard, ECM components play a critical role in reproductive performance (15). An abnormal turnover of ovarian ECM components has been considered in PCOS patients in a previous report (35). Of the altered genes, downregulation of *COL1A1* and *FN1* in IS patients in addition to *LAMA1* and *LAMA2* in IR versus IS patients was not previously reported. To the best of our knowledge, the current study was the first real time based simultaneous analysis of more than 80 ECM and cell adhesion genes as a more reliable technique compared to microarrays. 

*ECM1* is a secretory glycoprotein which regulates cell proliferation and invasion by an increase in glucose transporter (GLUT) expression (36). In this study, we found that the *ECM1* gene downregulated in the IR group. Since glucose is necessary for oocyte maturation, *ECM1* downregulation could reflect the role of IR in antral follicle arrest in PCOS patients.

Integrin (ITG) families are heterodimeric integral membrane proteins composed of an alpha subunit and a beta subunit that function in cell surface adhesion and signaling (37). According to the results by Liu et al. (38), the ITG gene family downregulated in PCOS cumulus cells compared with a control group. Due to the importance of ITG genes in cell adhesion, they suggested that the communication of oocyte and its neighboring cumulus cells in PCOS patients might be disrupted. According to our data, *ITGA5* and *ITGA7* downregulated in PCOS patients compared to the control group. *ITGA7* functions as receptor for the basement membrane protein laminin-1. *ITGA5* is known as a fibronectin receptor. Recent studies have shown that alterations of some genes are associated with oocyte nuclear maturation in PCOS (39). Cell-matrix adhesion molecules such as ITG family are important in this process.

## Conclusion

Downregulation of ECM and cell adhesion molecule genes in cumulus cells of infertile PCOS women with and without insulin resistance can have an association with decreased numbers of mature oocytes and good quality embryos.
